# InterCellDB: A User‐Defined Database for Inferring Intercellular Networks

**DOI:** 10.1002/advs.202200045

**Published:** 2022-06-02

**Authors:** Ziyang Jin, Xiaotao Zhang, Xuejiao Dai, Jinyan Huang, Xiaoming Hu, Jianmin Zhang, Ligen Shi

**Affiliations:** ^1^ Department of Neurosurgery Second Affiliated Hospital School of Medicine Zhejiang University Hangzhou Zhejiang 310058 China; ^2^ Department of Neurology First Affiliated Hospital School of Medicine Zhejiang University Hangzhou Zhejiang 310058 China; ^3^ Biomedical Big Data Center First Affiliated Hospital School of Medicine Zhejiang University Hangzhou Zhejiang 310058 China; ^4^ Pittsburgh Institute of Brain Disorders and Recovery and Department of Neurology School of Medicine University of Pittsburgh Pittsburgh PA 15213 USA

**Keywords:** cellular localization, functional annotation, intercellular network, scRNA‐seq, user‐defined database

## Abstract

Recent advances in single cell RNA sequencing (scRNA‐seq) empower insights into cell–cell crosstalk within specific tissues. However, customizable data analysis tools that decipher intercellular communication from gene expression in association with biological functions are lacking. The authors have developed InterCellDB, a platform that allows a user‐defined analysis of intercellular communication using scRNA‐seq datasets in combination with protein annotation information, including cellular localization and functional classification, and protein interaction properties. The application of InterCellDB in tumor microenvironment research is exemplified using two independent scRNA‐seq datasets from human and mouse and it is demonstrated that InterCellDB‐inferred cell–cell interactions and ligand–receptor pairs are experimentally valid.

## Graphical abstract

User‐defined analysis: A platform is developed for user‐defined analysis on intercellular crosstalk using single cell RNA sequencing (scRNA‐seq) datasets. Protein annotation including expression change, cellular localization and functional classification are considered. Protein interaction properties including evidence source, credibility score, action mode, and action effect are provided as well. Therefore, biological meaningful processes can be explored further by InterCellDB.

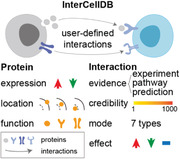

## 1 Introduction

Cell crosstalk with adjacent or remote partners are commonly observed in multicellular organisms throughout the life span.^[^ ^1^ ^]^ Continual cell–cell interactions ensure the coordination of cellular activities to maintain physiological homeostasis.^[^ ^2^ ^]^ Under disease conditions, intercellular networks change dramatically due to the alterations in the extracellular matrix.^[^ ^3^ ^]^ For instance, tumor cells modify the surrounding micro‐environment by secreting factors to recruit immune cells, which in turn impact on tumor growth and its responses to therapies.^[^ ^4^ ^]^ Elucidating intercellular communications will enrich our knowledge of disease development and promote new therapeutic approaches.

Single‐cell‐omics technologies provide sufficient resolution to map intercellular networks.^[^ ^5^ ^]^ Several analytical tools have been developed to unravel intercellular interactions, such as CellPhoneDB,^[^ ^6^ ^]^ SingleCellSignalR,^[^ ^7^ ^]^ CellChat,^[^ ^8^ ^]^ iTALK,^[^ ^9^ ^]^ and NicheNet.^[^ ^10^ ^]^ However, there are still unmet needs in this field. First, all current analytical tools provide limited types of interaction. In reality, intercellular networks are much more complicated, encompassing ligand–receptor, receptor–receptor, extracellular matrix–receptor, and vesicle–cell interactions.^[^ ^1^ ^]^ Besides, all these published tools have limited capability in providing insight in intercellular interactions for specific functions. For instance, in recent study, we found no available analysis approaches able to predict how ligand–receptor interaction between regulatory T cells (Treg cells) and microglia promotes oligodendrogenesis and white matter repair after ischemic stroke.^[^ ^11^ ^]^ Finally, most current approaches have fixed build‐in searching criteria, which limited the dimensions of analysis. A new platform is in demand to analyze intercellular networks in a user‐defined manner. We, therefore, develop a new analytical platform called InterCellDB to address these gaps and provide a user‐defined comprehensive analysis of intercellular communication using single cell RNA sequencing (scRNA‐seq) datasets in combination with specific biological functions of interest.

## 2 Results

### 2.1 Building and Running InterCellDB

**Figure 1:**
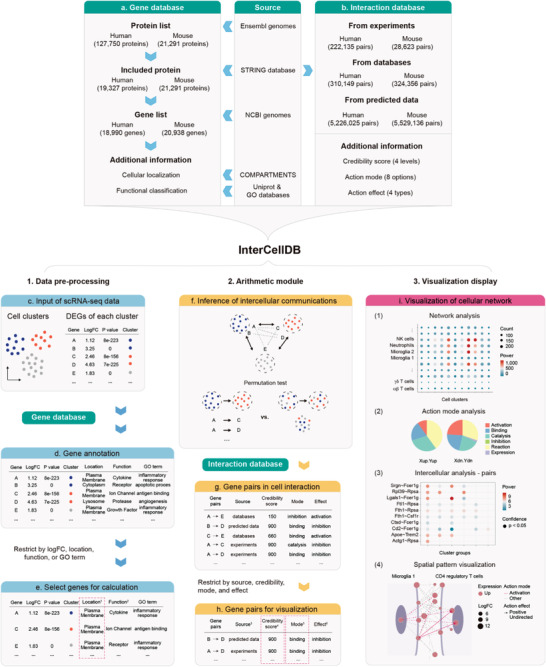
Overview of InterCellDB database and workflow. a,b) InterCellDB integrates public databases to generate analytic tools. c–i) Routine workflow of InterCellDB: data preprocessing (c–e), arithmetic module (f–h), and visualization display (i). It starts from differentially expressed genes (DEGs) and corresponding cell clusters annotation (c). All the DEGs were annotated with their cellular localization, functional classification, and their attribution in GO terms (d). Customized DEGs were selected by setting three modules, including gene expression, cellular localization, and functional feature (e). Permutation test was performed to calculate the confidence of interaction (f). Each gene pair was annotated with evidence sources, credibility score, action mode, and action effect by matching to the interaction database (g). Customized gene pairs were generated by setting evidence source, credibility score, action mode, and action effect for further visualization (h). Visualization of cellular network (i) including network analysis (i(1)), action mode analysis (i(2)), intercellular analysis (i(3)), and spatial pattern visualization (i(4)). ^1^ “Location” annotated genes with 13 types of cellular localization and the red dashed box showed the example for selecting proteins located in plasma membrane. ^2^ “Function” annotated genes with 132 types of functional features. ^3^ “Source” provided evidence sources including experimentally validated, pathway curated, and predicted. ^4^ “Credibility score” ranged from 1 to 1000 (larger means more credible) and had four confidence levels by cutting off at 400/700/900. The red dashed box showed the example for selecting proteins with credibility score 900. ^5^ “Mode” was action mode, referring to functional interplay between two proteins. The red dashed box showed the example for selecting proteins with action mode of binding. ^6^ “Effect” was action effect, referring to expression changes caused by one protein when it interacted with another protein.

Information from several public biological sources (Table S1, Supporting Information) was integrated to ensure the comprehensiveness, accuracy, and credibility of our dataset. As outline in **Figure** **1**, two major databases, including gene (Figure 1a) and interaction (Figure 1b) databases, were constructed to provide a customizable platform for intercellular network analysis. The gene database contained 18 990 human genes and 20 938 mouse genes with a variety of annotations, including cellular localization (Table S2, Supporting Information) and functional classification (Table S3, Supporting Information). Briefly, we first acquired the complete human (127 750 proteins) and mouse (21 291 proteins) protein data from the Ensembl genomes database (Figure 1a). Since not all proteins are involved in cell–cell interactions, we aligned the above protein lists with molecules involving protein–protein interactions in the STRING database (Figure 1a).^[^ ^12^ ^]^ Subsequently, based on the references provided by the NCBI genomes database, we converted proteins into corresponding coding genes and generated two gene lists (Human: 18 990 genes, Mouse: 20 938 genes; Figure 1a). Finally, we appended annotation information for each protein including cellular localization from the COMPARTMENTS database and functional classification from the Uniprot and Gene Ontology (GO) databases (Figure 1a).^[^ ^13^, ^14^, ^15^, ^16^ ^]^ To construct interaction database, we extracted all the information of protein–protein interaction from the STRING database. To meet the requirements of intercellular network analysis in various biological processes, we also provided the evidence source, credibility score, action mode, and action effect for each protein–protein interaction (Figure 1b).

InterCellDB starts with the fold changes and adjusted *p* values of the differentially expressed genes (DEGs, so‐called signature genes) in each cluster compared with other clusters (Figure 1c). All the DEGs were annotated by matching to the gene database, including their cellular localization, functional classification, and their attribution in GO terms (Figure 1d). It allows users to select specific genes for further calculation by setting three modules, including gene expression, cellular localization, and functional feature (Figure 1e). In addition, users can create their own lists of DEGs by selecting specific GO terms. For instance, to investigate how Treg cells released factors impact on microglia in an injured brain, the custom settings of the DEGs could be set as follows: 1) gene expression: up‐regulated DEGs of Treg cells and microglia; 2) cellular localization: extracellular region for Treg cells, and plasma membrane for microglia; 3) functional features: cytokine and trophic factor for Treg cells, and receptor for microglia. After data preprocessing, permutation test is performed to calculate the confidence of interaction (Figure 1f). Subsequently, the statistically significant gene pairs were matched to the interaction database. Only matched gene pairs were considered as potential interactions between two target cells. In addition, each gene pair was annotated with evidence sources, credibility score, action mode, and action effect (Figure 1g). It allows users to set these modules to select specific interactions for further visualization according to study requirements (Figure 1h).

Data calculation and visualization are available in InterCellDB by running the R software. InterCellDB performs multilayer calculations and display, including network analysis, action mode analysis, intercellular analysis, and spatial pattern visualization (Figure 1i). The network analysis defines the power of crosstalk between any two cell clusters (Figure 1i(1)). The dot size represents the total count of protein–protein interactions involving in crosstalk between two types of cells. The dot color suggests the significance of intercellular communications, of which the computational formula is described in the Experimental Section. The intercellular analysis assesses action effect and mode of action between two groups of cells (Figure 1i(2)). There are several other custom visualization options including maps of the particular protein–protein pairs in all intercellular networks (Figure 1i(3)), and spatial pattern of proteins involving in cell–cell crosstalk (Figure 1i(4)). With the aid from InterCellDB, many previously manually performed cell–cell interaction analysis using different experimental systems could be replaced by computerized analysis using single cell datasets collected from targeted tissues, which is more accurate and less time and labor consuming.

### 2.2 Applications of the InterCellDB

The InterCellDB can be applied to any scRNA‐seq dataset containing potentially interacting cell populations from human or mouse. Here, we describe in detail how InterCellDB can be utilized to explore intercellular crosstalk in two scenarios.

**Figure 2:**
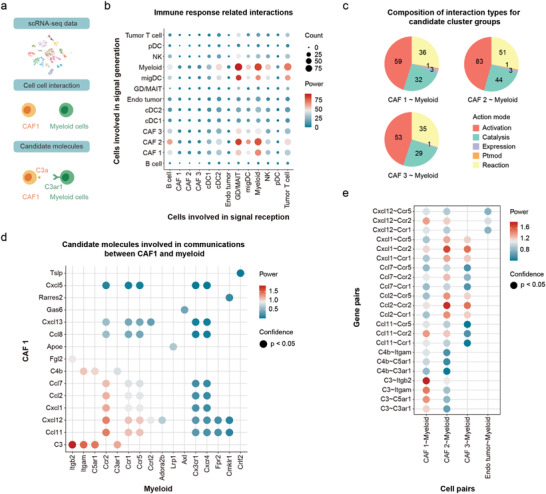
Analysis by InterCellDB on mouse melanoma scRNA‐seq data. a) Schematic illustration of analysis processes, which starts from cluster information collected from the scRNA‐seq datasets. Candidate 2‐cell interactions between CAF1 and myeloid cells are evaluated. Finally, the target gene pair is identified. b) Dot plot showing network analysis on immune response related genes. All statistically significant interactions (*p*‐value < 0.05 in permutation test) are fetched. Dot size shows the count of gene pairs. The color of dots indicates the aggregated strength of interaction. c) Pie plot showing the composition (in count) of action modes between the subsets of cancer‐associated fibroblasts (CAF1, CAF2, and CAF3) and the myeloid cells. d) Dot plot showing the comparison on candidate gene pairs (*p*‐value < 0.05) between CAF1 and myeloid cells. e) Dot plot showing the selected genes pairs along with all their co‐occurring interactions. Gene pairs (*p*‐value < 0.05) are included. Dot color represents the power of gene pairs by multiplying the expression levels of participating genes. pDC, plasmacytoid dendritic cell; NK, natural killer cell; migDC, migratory dendritic cell; GD/MAIT, mucosal‐associated invariant T cell; Endo tumor, tumor endothelial cells; cDC1/2, conventional dendritic cell; CAF 1/2/3, cancer‐associated fibroblast. Ptmod, posttranslational modification.

In the first study, we applied InterCellDB to mouse scRNA‐seq data generated by Davidson et al. to study tumor microenvironment in melanoma. CellPhoneDB database of receptor–ligand interactions was used in the original publication to explore immune–stromal interactions.^[^ ^17^ ^]^ The result highlighted a significant crosstalk between a subset of cancer‐associated fibroblasts (CAF1) and myeloid cells (**Figure** **2**a). Moreover, Davidson et al. predicted and proved that the CAF1 produces C3a to recruit C3aR^+^ myeloid cells into tumor mass and promote tumor growth (Figure 2a).

We applied InterCellDB on Davidson's dataset for further intercellular network analysis with a focus on genes related to immune response (*GO: 0 006955*). According to the function and cellular localization of proteins, we classified two core collections of cells: 1) cells releasing signals (i.e., cells with proteins released to the extracellular region); 2) cells receiving signals (i.e., cells with receptors expressed in the plasma membrane). Intercellular network analysis between these two collections of cells showed that myeloid cells were one of the main signal recipients in response to the ligands from CAF1, CAF2, and myeloid cells (Figure 2b). We then performed intercellular analysis to evaluate whether CAF1, CAF2, or CAF3 was the main candidate cell group to recruit myeloid cells. Similar interaction mode was observed among these three populations (Figure 2c). The rank of all protein pairs between CAF1 and myeloid cells identified several chemokine–receptor pairs including C3–Itgb2, C3–Itgam, C3–C5ar1, C3–C3ar1, and Ccl11–Ccr2 as candidate molecules that mediate CAF1‐myeloid cell interaction (Figure 2d). Finally, we evaluated whether these candidate protein pairs are specific for CAF1‐myeloid cell interaction or also participated in intercellular crosstalk between other cells (Figure 2e). We found that the C3–Itgb2, C3–Itgam, C3–C5ar1, and C3–C3ar1 pairs were relatively specific for CAF1–myeliod cell interaction and should be good candidates for further biological studies (Figure 2e). Interestingly, biological experiments in Davidson's study prove that C3–C3ar1 pair plays critical roles in intercellular communications between CAF1 and myeloid cells.

To compare the results of cellular interaction analysis using InterCellDB with those using other published tools, we performed intercellular analysis between CAF1 and myeloid cells (Figure S1a, Supporting Information). Comparable candidate interactions were predicted by InterCellDB, CellPhoneDB, NicheNet, and CellChat (Figure S1a, Supporting Information). Among those 15 protein pairs predicted by InterCellDB, nine of them (60%) were also identified by the other three tools (Figure S1b, Supporting Information). Subsequently, we calculated the percentage of protein pairs that are supported by previous literatures among all the predicted protein pairs by InterCellDB and other tools (Figure S1c, Supporting Information). The predicted accuracy of InterCellDB (86.6%) was higher than those of the other three tools (75%) (Figure S1c and Table S4, Supporting Information). Together, InterCellDB showed a superior value in prediction of cellular interaction to other previous tools, including CellPhoneDB, NicheNet, and CellChat.

**Figure 3:**
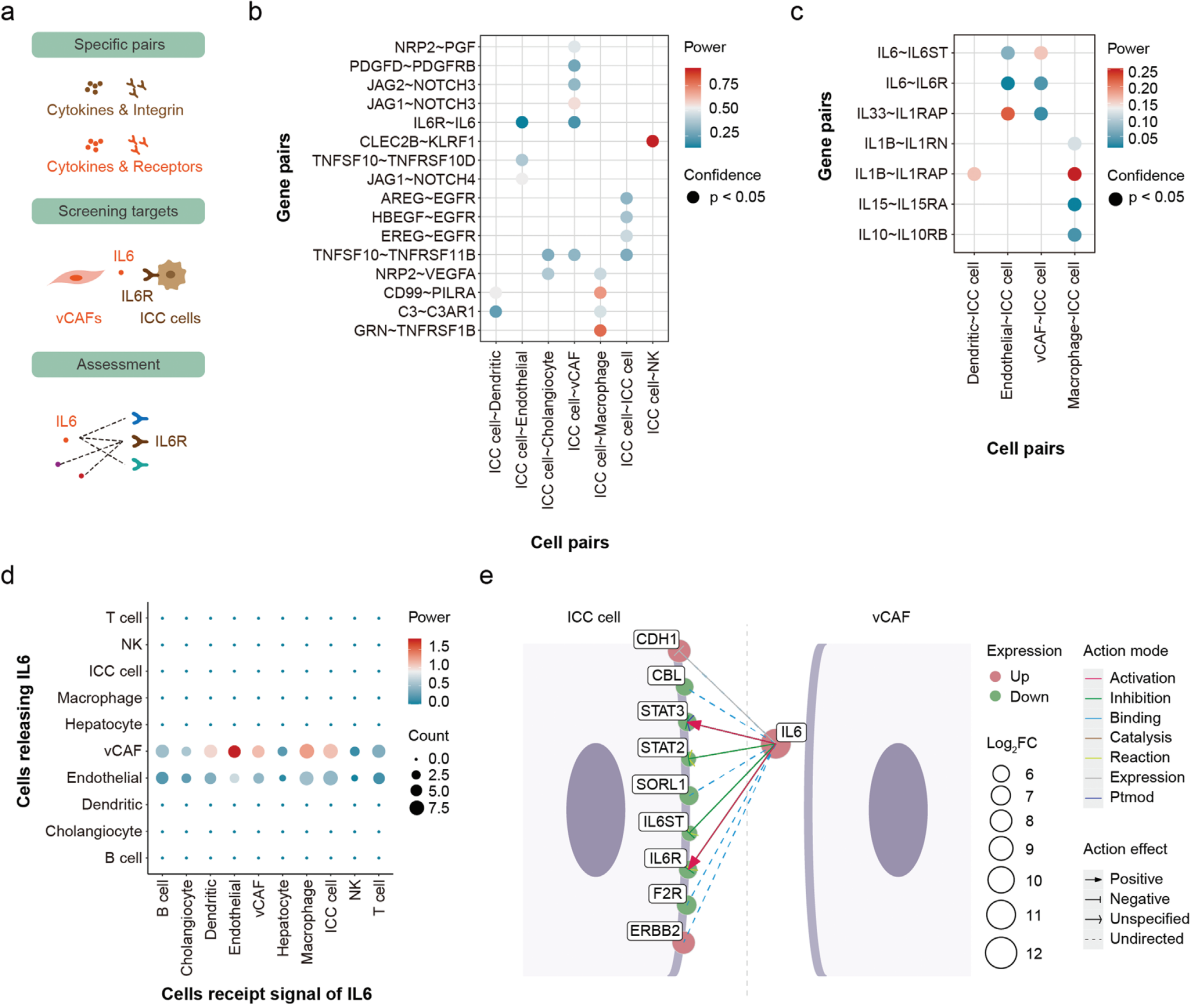
Figure Analysis of off‐target effects by InterCellDB on human intrahepatic cholangiocarcinoma (ICC) scRNA‐seq data. a) Schematic illustration of processing steps. Zhang et al. screened several gene pairs and finally identified IL6–IL6R interaction between tumor cells and niche cells. To evaluate potential off‐target effects, we assessed all gene pairs involving either IL6 or IL6R. b) Gene pairs identified by Zhang et al. are reanalyzed using InterCellDB. c) Dot plot showing result of evaluation on interleukin‐related gene pairs. Gene pairs with *p*‐value < 0.05 in permutation test are fetched. Dot color represents the power of gene pairs by multiplying the expression levels of participating genes. d) Dot plot showing analysis of network involving IL6. Dot size and color indicate the count and aggregated strength of interaction respectively. e) All IL6‐related gene pairs between vCAF and ICC cell. Interaction type and effect are denoted by line color and arrow, respectively. Genes are drawn as dots, and gene expression changes are indicted by size. The up‐regulated genes are denoted as red and down‐regulated genes are denoted as green. vCAF, vascular cancer‐associated fibroblast; NK, natural killer cell. Ptmod, posttranslational modification.

InterCellDB can also be applied to evaluate the off‐target effects of a specified protein pair in the intercellular network. This complementary type of analysis reminds researchers to pay attention to the side effects of knocking out or knocking down a specific gene in the intercellular communication network. To exhibit this application of InterCellDB, we provided a second case of analysis using human scRNA‐seq data generated by Zhang et al., which studies intercellular crosstalk between tumor cells and niche cells in the intrahepatic cholangiocarcinoma (ICC).^[^ ^18^ ^]^ Zhang et al. performed scRNA‐seq and identified vascular cancer‐associated fibroblasts (vCAFs) with high levels of IL6 as the most prominent cluster among six distinct fibroblast subsets. Their biological studies proved that vCAFs secrete IL6 to induce significant epigenetic alterations in ICC cells expressing IL6 receptors and thereby enhance malignancy (**Figure** **3**a).

We applied InterCellDB to a group of ligand–receptor pairs specified in Zhang's analysis and evaluated whether IL6–IL6R was the potential messenger between vCAFs and ICC cells (Figure 3b). The results showed a similar strength distribution to the calculation by CellPhoneDB (Figure 3b). Several protein pairs including IL6–IL6R, NOTCH3–JAG1, NOTHCH3–JAG2, PDGFD–PDGFRB, and PGF–NRP2 were mainly involved in intercellular communications between vCAFs and ICC cells (Figure 3b). These protein pairs were also predicted by other tools, including CellPhoneDB, NicheNet, and CellChat (Figure S1d, Supporting Information). Furthermore, our analysis suggested NOTCH3–JAG1 pair as a recommended protein pair that mediates vCAFs, and ICC cells interaction. And IL6–IL6R pair was also a potential candidate according to our analysis, which was verified by biological experiments in Zhang's study. Subsequently, we extracted all interleukins and their receptors from our databases to assess whether there were other potential candidates in addition to the IL6–IL6R pair. We found that only IL6‐mediated and IL33‐mediated protein pairs were involved in cell‐to‐cell communication between vCAFs and ICC cells (Figure 3c). This result was also observed in the prediction of cellular interaction by other tools, including CellPhoneDB, NicheNet, and CellChat (Figure S1e, Supporting Information). We further evaluated the off‐target effects of IL6–IL6R pair in the intercellular network (Figure 3d). Interestingly, vCAFs and endothelial cells produced IL6 could act on multiple cell types, including endothelial cells, macrophages, and ICC cells (Figure 3d). Therefore, the knockout of IL6 gene in mice may have effects on not only ICC cells, but endothelial cells and macrophages as well. Finally, InterCellDB provided spatial distribution map of IL6‐mediated protein–protein pairs encompassing a wide variety of information, such as the expression of proteins, action mode, action effect, and cellular localization (Figure 3e).

### 2.3 Comparison with Other Published Methods

The major differences between InterCellDB and other published methods including CellChat, iTALK, CellPhoneDB, SingleCellSignalR, and NicheNet are summarized (Table S5, Supporting Information). First, most published methods calculate intercellular crosstalk based only on ligand–receptor pairs, but ignore other common types of interactions including receptor–receptor, extracellular matrix–receptor, and vesicle–cell interactions. Indeed, the annotation of protein functions is insufficient for these previous methods. Only the iTALK divides ligands into several functional subsets, including cytokine, growth factor, immune checkpoint, and others.^[^ ^9^ ^]^ Distinct from other analytical tools, InterCellDB aggregates all gene‐coded proteins into 132 types according to the annotated information from the Uniprot database (Table S3, Supporting Information). To facilitate use of InterCellDB, we further categorized these 132 types into 16 classifications, including cytokine, receptor, enzyme, etc. (Table S3, Supporting Information). Hence, InterCellDB could provide multiple patterns of analysis by designating the types of proteins, such as cytokine–receptor, and growth factor–integrin. Second, it is known that not all protein pairs are involved in intercellular communication. For instance, an intracellular enzyme in one cell is not likely to directly act on a translation regulator in the nucleus of another cell. To filter out biologically unlikely interactions, we incorporated the cellular localization information of all gene‐encoded proteins from the COMPARTMENTS database, which provides protein subcellular localization with confidence scores based on integrated analysis of multiple databases and prediction tools (Table S2, Supporting Information). As one protein may appear in multiple locations in a cell, we therefore retained the information of confidence scores on cellular localization for each protein. Initial credibility of cellular localization is recommended to set as four and five. Users can adjust this cut‐off value depending on their experimental requirements. Finally, InterCellDB predicts the action mode and effect of protein–protein interactions. Action mode contains eight options including activation, inhibition, catalysis, and expression, post‐translational modification, binding, reaction, and other (Table S6, Supporting Information). Action effect refers that one protein influences the expression of another protein (Table S7, Supporting Information). All the above‐mentioned novel features ensure a customizable cellular network analysis with high biological relevance and credibility.

**Figure 4:**
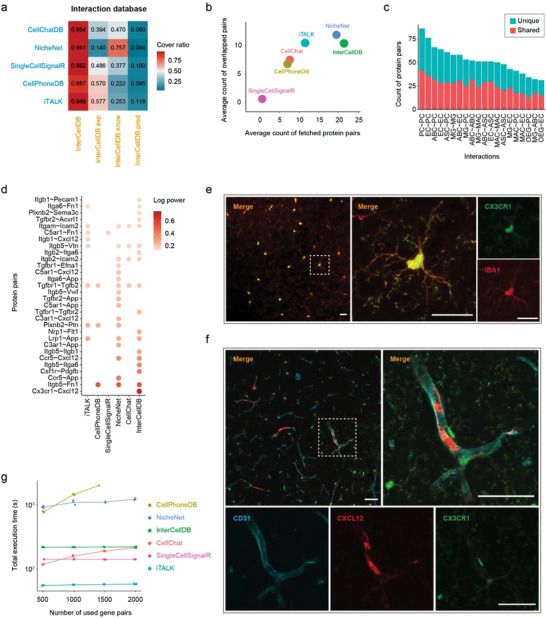
Comparison between InterCellDB and other analytical tools. a) The coverage ratios of gene pairs generated by InterCellDB to the gene pairs from other methods. The whole database is split to three sub‐databases: InterCellDB.exp: experimentally validated gene pairs, InterCellDB.know: pathway curated gene pairs, InterCellDB.pred: predicted gene pairs. b–e) we employed scRNA‐seq data on mouse brain as a testing dataset to evaluate the breadth and accuracy of inference results by InterCellDB comparing with the other methods. b) The breadth of inference results by InterCellDB comparing with other methods. The *x*‐axis indicates the average count of predicted protein pairs by one method, and the *y*‐axis indicates the average number of consistently predicted protein pairs among these methods. c) Count of predicted protein pairs by InterCellDB and other methods. Red bar denotes the number of protein pairs predicted by InterCellDB and at least one of other methods. Blue bar denotes the count of those uniquely identified by InterCellDB. d) Top predicted protein pairs by any of these methods for crosstalk between microglia and endotheliocytes. e,f) Representative confocal micrographs showing the brain slices of CX3CR1‐GFP mice. Scale bars = 40 µm. e) Red: IBA1 positive cells, Green: CX3CR1 positive cells. f) CX3CR1 positive cells (Green) locate near the CXCL12‐expressing CD31 positive cells (colocalization of Red and Cyan). g) Running time of all methods under the same settings. ABC, arachnoid barrier cells; ASC, astrocytes; EC, endothelial cells; MAC, macrophages; MG, microglia; OEG, olfactory ensheathing glia; and PC, pericytes.

In order to better comparing with those databases of previous tools, we performed several comprehensive benchmark studies. First, we matched protein pairs explicitly between InterCellDB and previous databases, which showed that InterCellDB covered about 90% of all protein pairs in other databases in human (**Figure** **4**a). Three major sub‐libraries including experiment validated (InterCelldDB.exp), pathway curated (InterCelldDB.know), and predicted databases (InterCelldDB.pred) were also incorporated into the comparison. Interestingly, the CellPhoneDB and iTALK better aligned to experiment‐validated pairs from the InterCellDB.exp database (Figure 4a). The NicheNet aligned better to pathway curated pairs from the InterCellDB.know database (Figure 4a). These comparisons revealed wide coverage, high fidelity, and low selection bias of InterCellDB. Second, we applied NicheNet and CellChat to analyze the above‐mentioned two cases on mouse melanoma (Figure 2), and human intrahepatic cholangiocarcinoma (Figure 3). Third, we employed another mouse scRNA‐seq data generated by Ximerakis et al.^[^ ^19^ ^]^ as a testing dataset to evaluate the performance by InterCellDB comparing with the other methods. The results indicated that the predicted breadth of InterCellDB is similar to NicheNet (Figure 4b). In addition, InterCellDB generated considerable larger number of interactions and meanwhile kept fairly consistent results with the other methods across all interacting cell clusters (Figure 4c). Subsequently, we screened potential interactions between endotheliocytes, and microglia, and found that several unique interactions were predicted by InterCellDB (Figure 4d). Biological studies were performed to verify that Cxcl12 and Cx3cr1 were involved in microglia–endotheliocyte communications (Figure 4e,f). Finally, the results of computational efficiency showed that InterCellDB ran much faster than NicheNet and CellPhoneDB, with a performance close to those methods not using statistical test during runtime (Figure 4g).

## 3 Discussion

To summarize, a major strength of InterCellDB, as compared with most other databases, is that it enables user‐defined analyses of intercellular crosstalk, and takes into account the function and cellular localization of proteins, the action mode and effect of protein–protein interaction. InterCellDB is applicable to any of human and mouse scRNA‐seq dataset containing potentially interacting cell populations. In addition, we include a complete list of all possible protein–protein interactions, which provides comprehensive information when interpreting cell–cell communication networks.

One limitation of InterCellDB is that it could not provide information about how intercellular interaction leads to downstream signaling events. Fortunately, the NicheNet offers a solution to predict the downstream signal transduction in the receiving cells after engaged by the ligands from sender cells. The combined use of InterCellDB with NicheNet may provide thorough analysis to picture a complete cell–cell interaction and signaling network.

In summary, InterCellDB provides a unique platform for customizable intracellular communication analysis taking into consideration of the subcellular localization, and biological functions of interacting partners. We expect that InterCellDB will be applied to elucidate the intercellular networks in health and disease states.

## 4 Experimental Section

### Construction of Gene‐Coded Protein Database

Information of gene‐protein correspondence was collected from the Ensembl and NCBI gene database on March 20, 2020 (Table S1, Supporting Information).^[^ ^20^ ^]^ Annotation information including subcellular location and molecular function was added for each gene‐coded protein (Tables S2 and S3, Supporting Information). Subcellular location was extracted from the COMPARTMENTS database.^[^ ^13^ ^]^ A total of 13 independent location parameters were annotated for each gene‐coded protein, including “extracellular region,” “plasma membrane,” “cytosol,” “cytoskeleton,” “peroxisome,” “lysosome,” “endoplasmic reticulum,” “Golgi apparatus,” “endosome,” “mitochondrion,” “cytoplasm,” “nucleus,” and “other” (Table S2, Supporting Information). Molecular function was collected from the Uniprot database.^[^ ^14^ ^]^ All 132 classifications in the “Molecular Function” panel of the Uniprot database were included in our present database. For the convenience of the user, we divided them into 16 independent groups including “actin‐binding and motor protein,” “antimicrobial,” “cytokine,” “enzyme,” “growth factor,” “hormone,” “hydrolase,” “ion channel,” “protein metabolism regulator,” “receptor,” “transcription regulator,” “transducer,” “transferase,” “translation regulator,” “vasoactive,” and “other” (Table S3, Supporting Information).

### Construction of Protein Interaction Database

Protein–protein interactions were collected from the STRING database (version 11, https://version‐11‐0.string‐db.org).^[^ ^12^ ^]^ Three subsets were constructed according to the credibility of protein interactions. The first subset contained those protein interactions that were validated by experiments. These protein interactions were labelled with “experiments > 0” in the STRING database. The second subset included those protein interactions that were extracted from pathways and labelled with “database > 0” in the STRING database. The last subset contained those protein interactions from various sources including “neighborhood,” “neighborhood_transferred,” “fusion,” “cooccurence,” “homology,” “coexpression,” “coexpression transferred,” “experiments transferred,” “database transferred,” “textmining,” and “textmining transferred,” which showed low evidence and were labelled with score > 0 in at least one of the above sources in the STRING database. We named these three subsets as experiment validated, pathway curated, and predicted databases. In addition, annotation information including credibility score, action mode, and action effect was added for each protein–protein pair. Credibility score ranging from 1 to 1000 are divided into four levels of confidence: highest (score ≥ 900), high (score ≥ 700), medium (score ≥ 400), and low (score < 400). Action mode refers to functional interplay between protein *x* and *y*, including “activation,” “binding,” “catalysis,” “expression,” “inhibition,” “post‐translational modification (ptmod),” “reaction,” and “other” (Table S6, Supporting Information). Action effect refers to expression changes of protein *x* influenced by protein *y*, which contains “positive,” “negative,” “unspecified,” “undirected” (Table S7, Supporting Information).

### Calculation of Interaction Power and Confidence

The power of interaction was calculated by the product of expressions of involved proteins. For one interacting protein pair *A* and *B*, the power was calculated by:

(1) $$\mathrm{Power}\left(A,B\right)=\mathrm{Exprs}\left(A\right)*\mathrm{Exprs}\left(B\right)$$

where *Exprs(A)* and *Exprs(B)* mean the normalized expression levels of the proteins.

To calculate the confidence of interaction, cell label permutation test was used to randomly assign clusters labels to all cells and recalculate the power of all interactions. The *p*‐value of one given interaction between protein *A* and *B* was calculated as:

(2) $$p=\frac{\left\{\#n\left|Power{\left(A,B\right)}^{\left(n\right)}\leq Power\left(A,B\right),n=1,2,\text{…},N\right.\right\}}{N}$$

where Power*(A, B)^(n)^* is the power of the interaction in *n*‐th permutation. *N* is the total number of permutations (*N* = 100 by default). Those interactions with *p*‐value < 0.05 are considered significant.

### Calculation of Interaction Intensity among Cell Types

The number of protein–protein pairs and aggregated power *W* between any two cells were calculated to evaluate the intensity of cell–cell interaction. The aggregated power *W* is the sum of interaction power for all protein pairs. Given two cell clusters *i* and *j*, the aggregated power was calculated by:

(3) $$W\left(i,j\right)=\sum_{k=1}^{M}Power\left(A_{k},B_{k}\right)$$

where *M* represents the overall count of protein pairs between cell cluster *i* and *j*. For *k*‐th interaction, the power of protein *A*_k_ and *B*_k_ are calculated.

### Customization of the Analysis Process

The major advantage of the InterCellDB was that it provided custom settings for specific analysis accommodating to complicated biological process. There were two main modules of custom settings, including the selection of proteins in the analysis and the restriction of functional scope. The users could set specified thresholds for expression levels, cellular localizations, and functional features to select appropriate proteins for analysis. The users could also restrict functional scope by setting the evidence sources, credibility score, action mode, and action effect of the protein interactions.

### Collection and Analysis of the Mouse scRNA‐seq Data from Davidson et al

Raw count files and experiment metadata were downloaded for scRNA‐seq data of mouse melanoma generated by Davidson et al. from https://www.ebi.ac.uk/gxa/sc/experiments/E‐EHCA‐2/downloads. scRNA‐seq data preprocessing was completed by Seurat (v3.2) before applying InterCellDB for intercellular analysis.^[^ ^21^ ^]^ First, the count matrix was correctly formatted by setting the row names as gene names and the column names as cell barcodes. Then, the data were normalized and scaled. Cell clusters were annotated by the references of the experiment metadata, which included cancer‐associated fibroblasts (CAF 1–3), fibroblast from lymph node (fibroblast LN), tumor endothelial cells (Endo tumor), lymphatic endothelial cell (Endo lymphatic), endothelial cells from lymph node (Endo LN), conventional dendritic cells (cDC 1–2), plasmacytoid dendritic cell (pDC), migratory dendritic cell (migDC), T cell from lymph node (LN T cell), mucosal‐associated invariant T cell (MAIT), tumor T cells, natural killer cell (NK), myeloid, and B cells. Further, *t*‐SNE dimensionality reduction was performed to visualize the result of clustering and the differentially expressed genes were calculated for all clusters.

### Assessment of the Magnitude of Immune Responses among Cell Types

The study performed by Davidson et al. aims to explore immune–stromal interactions. Hence, the functional scope was restricted within immune response. Briefly, 1671 unique genes were extracted from the GO term of immune response (*GO: 0 006955*). All further analysis was restricted in this cluster of genes. Cells with proteins releasing to the extracellular region were set as *y*‐axis and cells with receptors expressing in the plasma membrane were set in *x*‐axis. Action mode of protein pairs was set as “binding” to ensure that factors released from cells of the *y*‐axis could bind to their receptors in cells of the *x*‐axis. Heatmap was used to present the interaction intensity. Dot size showed the count number of protein pairs, and the color of dots indicated the strength of interaction.

### Screening Candidate Molecules Involved in Communications between CAF1 and Myeloid Cells

To screen out candidate molecules participating in the activation of myeloid cells by CAF1, all protein pairs labelled with “activation” (action mode) and “positive” (action effect) between CAF1 and myeloid cells were extracted. Heatmap was used to present the intensity and confidence of protein pairs in communications between CAF1 and myeloid cells. Dot size represented the confidence of protein pairs. The degree of color saturation of a dot represented the power of protein pairs by summing up the log_2_ fold changes of participating proteins.

### Collection and Analysis of the Human scRNA‐seq Data from Zhang et al

Raw gene expression count matrix of human ICC scRNA‐seq data were downloaded from GEO with accession code GSE138709. To determine gene expression, the same version of Seurat (v2.3.4) as described in the original study was applied. After normalization, 1000 variable genes were selected for cell clustering (resolution: 0.3). Cell clusters were annotated by the references of the experiment metadata as *T* cells, NK cells, malignant cells, macrophages, hepatocytes, fibroblasts, endothelial cells, dendritic cells, cholangiocytes, and *B* cells. Further, *t*‐SNE dimensionality reduction was performed to visualize the result of clustering and the differentially expressed genes for all clusters were calculated.

### Evaluation of the Off‐Target Effects of a Specified Protein Pair in the Intercellular Network

To evaluate potential off‐target effects, all protein pairs involving either IL6 or IL6R were assessed. Briefly, a set of ligand–receptor pairs predicted by Zhang et al. was extracted and it was reanalyzed by using InterCellDB. Heatmap showed the intensity and confidence of these ligand–receptor pairs for all possible interactions among any two cell types. All interleukin receptors that were expressed in malignant cells and had at least one putative ligand expressed in the other cell types were calculated to screen candidate ligand–receptor pairs. Finally, IL6‐released cells were set in *y*‐axis and IL6‐received cells were set in *x*‐axis. Action mode of protein pairs was set as “binding” to ensure that factors released from cells of the *y*‐axis could bind to their receptors in cells of the *x*‐axis. Dot size and color indicated the count and strength of interaction, respectively.

### Spatial Pattern of Target Protein Pairs between Two Cells

The spatial pattern of target protein pairs was shown in a schematic diagram. Two separate cell‐mimic areas represented two independent cells. Each cell area contained four subcellular locations, including extracellular space, cytomembrane, cytoplasm, and cell nucleus. Interaction type and effect were denoted by line color and arrow. Proteins were drawn as dots, and gene expressions were indicted by size. The up‐regulated genes were denoted as red and downregulated genes were denoted as green.

### Comparison of Protein Interaction Database between InterCellDB and Previous Methods

The coverage of protein interactions was assessed among InterCellDB, iTALK, CellPhoneDB, SingleCellSignalR, NicheNet, and CellChatDB. Three major subsets of InterCellDB including experiment validated (InterCelldDB.exp), pathway curated (InterCelldDB.know), and predicted databases (InterCelldDB.pred) were also incorporated into the comparison. The formula for coverage ratio for previous database *i* is given as:

(4) $$\mathrm{Cove}\mathrm{rage}\mathrm{ratio}\left(i\right)=\frac{\left|\mathrm{Pairs}\left(\mathrm{Inte}\mathrm{rCel}\mathrm{lDB}\right)\cap \mathrm{Pairs}\left(i\right)\right|}{\left|\mathrm{Pairs}\left(i\right)\right|}$$

where *Pairs(InterCellDB)* is protein pairs from InterCellDB, and *Pairs(i)* is protein pairs in previous database *i*.

### Comparison of Results between Methods on Mouse and Human Cases

For case on mouse melanoma, the interaction between CAF1 and myeloid cells by CellPhoneDB, NicheNet, CellChat, and InterCellDB was analyzed. To make the number of interactions comparable, the interactions between two differentially expressed genes with log_2_fold change > 0.25 and belonging to genes associated with immune response (*GO: 0 006955*) were preserved. To compare the precision between methods, literatures about these gene pairs were manually collected (Table S4, Supporting Information). One gene pair was defined as literature support if this interaction was validated to be associated with carcinogenesis or anti‐tumor function by experiment. For case on human cholangiocarcinoma data, similar analysis given in Figure 3b,c by CellPhoneDB, NicheNet, CellChat was performed and the results were compared with InterCellDB.

### Comparison of Performance between InterCellDB and Previous Methods

To compare the performance across all methods, a testing protein dataset was generated that only included those proteins existing in all databases (Table S8, Supporting Information). Using this dataset, the crosstalk between two cell clusters was calculated based on the interaction database for each of the methods. The scRNA‐seq data for mouse brain was downloaded from GEO repository with accession ID: GSE129788 as a testing dataset.^[^ ^19^ ^]^ Total 25 cell clusters were annotated by the references of the experiment metadata. Cell clusters from “young” animals and counted over 100 were used, which were given as arachnoid barrier cells (ABC), astrocytes (ASC), endothelial cells (EC), ependymocytes (EPC), immature neurons (ImmN), macrophages (MAC), microglia (MG), mature neurons (mNEUR), neuroendocrine cells (NendC), neural stem cells (NSC), olfactory ensheathing glia (OEG), oligodendrocytes (OLG), oligodendrocyte precursor cells (OPC), and pericytes (PC). The potentially interacting protein pairs between any two cell clusters were calculated by InterCellDB or any previous methods. The average count of fetched protein pairs for one method *k* was given as:

(5) $$\mathrm{Coun}t_{k}=\frac{\sum_{i=1}^{N}\sum_{j=1}^{N}\left|\mathrm{Pai}r_{i,j}\left(k\right)\right|}{N*N}$$

where *N* is the total number of cell clusters, and interactions generated by method *k* between cell cluster *i* and cell cluster *j* is given as *Pair_i,j_(k)*.

One fetched protein pair for one method could be defined as overlapped to the other if it could be validated by at least one of other methods. The average count of overlapped protein pairs for one method *k* are given as follows:

(6) $$\mathrm{Over}\mathrm{lapp}\mathrm{edco}\mathrm{un}t_{k}=\frac{\sum_{i=1}^{N}\sum_{j=1}^{N}\left|\mathrm{Pai}r_{i,j}\left(k\right)\cap {⋃}_{l=1,l\neq k}^{M}\mathrm{Pai}r_{i,j}\left(l\right)\right|}{N*N}$$

where *N* is the number of all included cell clusters, *M* is the number of overall candidate methods, and interactions generated by method *k* and *l* between cell cluster *i* and cell cluster *j* is given as *Pair_i,j_(k)* and *Pair_i,j_(l)*, respectively.

Details about runtime parameters and the version of each method are given in Note S1 (Supporting Information).

### Comparison of Computational Efficiency between InterCellDB and Other Methods

The scRNA‐seq data for mouse brain (GSE129788) was used a testing dataset.^[^ ^19^ ^]^ The analysis of computational efficiency was performed in Linux on a computer with 2.70 GHz Intel(R) Core (TM) i7‐10850H CPU, and 64.0 GB RAM. All programs were run in one core for three repeats. In order to be comparable, these methods were tested using the same number of protein pairs which set as 500, 1000, 1500, and 2000. Runtime were measured with “/user/bin/time ‐v” command in Linux.

### Animals

Male C57BL/6 CX3CR1‐GFP mice (8–10 weeks old) were housed in plastic cages with controlled temperature and humidity and a 12/12 h light/dark cycle. All animal experiment protocols were approved by the Institutional Ethics Committee of the Second Affiliated Hospital, Zhejiang University School of Medicine and were in accordance with the Guide for the Care and Use of Laboratory Animals of the National Institutes of Health.

### Immunostaining of Brain Sections

Mice were deeply anesthetized and perfused transcardially with 25 mL of ice‐cold phosphate‐buffered saline (PBS), followed by 20 mL of 4% paraformaldehyde (PFA). Brains were post‐fixed in 4% PFA for 24 h and dehydrated in serial 15% and 30% sucrose solutions at 4 °C. Next, the brain samples were sectioned into 25 µm thick coronal slices. The sections were stored in cryoprotectant (40% PBS, 30% glycerol, 30% ethylene glycol) and kept at −20 °C until immunostaining. Brain sections were washed twice with PBS, followed by permeabilization in 0.5% Triton X‐100 at room temperature. Next, brain sections were blocked with 5% normal donkey serum in PBS for 1 h at room temperature and incubated overnight at 4 °C with the following primary antibodies: anti‐CD31 (Santa Cruz, sc18916, 1:50), anti‐IBA1 (Abcam, ab5076, 1:250), anti‐CXCL12 (Santa Cruz, sc74271, 1:50). The sections were then incubated in the dark with donkey secondary antibody conjugated with Alexa Fluor 555 or 647 (Invitrogen, 1:500) at room temperature for 1 h. After washing with PBS for three times, the sections were mounted on glass slides with mount‐G containing DAPI (Yeasen Biotech). Sections were observed and analyzed with a Leica TCS SP8 confocal microscope (Leica Microsystems). Images were adjusted for brightness and contrast using Fiji 2.1.0/1.53c. All confocal images were represented as maximum intensity projections.

### Result Presentation

The results of InterCellDB analysis were displayed in both graphs and tables. The graphs were drawn by using *Tool.ShowGraph*. The tables were output by using integrated function named *Tool.WriteTables*.

### Data Availability

The scRNA‐seq data for mouse melanoma was downloaded from https://www.ebi.ac.uk/gxa/sc/experiments/E‐EHCA‐2/downloads. The scRNA‐seq data for human intrahepatic cholangiocarcinoma was downloaded from the Gene Expression Omnibus (GEO) repository with accession ID: GSE138709. The scRNA‐seq data for mouse brain was downloaded from GEO repository with accession ID: GSE129788.

### Code Availability

InterCellDB is publicly accessible as an R package in GitHub (https://github.com/ZJUDBlab/InterCellDB). All the code and data used for processing of data sources and following analysis are available in figshare, whose links are given as follows:

1) Database generation
  a) Mouse: https://doi.org/10.6084/m9.figshare.17057342
  b) Human: https://doi.org/10.6084/m9.figshare.17057339
2) Comparison of InterCellDB and previous methods (Figure 4 related codes):
  a) Database comparison: https://doi.org/10.6084/m9.figshare.17057357
  b) Performance comparison: https://doi.org/10.6084/m9.figshare.17057525
3) Case studies on human and mouse data
  a) Human example data processing (Figure 3 and S1 related codes): https://doi.org/10.6084/m9.figshare.17029997
  b) Mouse example data processing (Figure 2 and S1 related codes): https://doi.org/10.6084/m9.figshare.17029985

## Conflict of Interest

The authors declare no conflict of interest.

## Supporting information

Supporting Information

Supplemental Table 2

Supplemental Table 3

Supplemental Table 4

Supplemental Table 8
