# Supplementary material for: InterCellDB: A User‐Defined Database for Inferring Intercellular Networks
Source: Adv Sci (Weinh). 2022 Jun 2;9(22):2200045. doi: 10.1002/advs.202200045 (PMC9353444; doi:10.1002/advs.202200045)
Supplement: Supplementary file 1 — Supporting Information [file ADVS-9-2200045-s002.pdf]

ADVANCED SCIENCE
Open Access

Supporting Information

for *Adv. Sci.*, DOI 10.1002/advs.202200045

InterCellDB: A User-Defined Database for Inferring Intercellular Networks

*Ziyang Jin, Xiaotao Zhang, Xuejiao Dai, Jinyan Huang, Xiaoming Hu, Jianmin Zhang* and Ligen Shi**

# Supporting Information

**InterCellDB: a user-defined database for inferring intercellular networks**

*Ziyang Jin, Xiaotao Zhang, Xuejiao Dai, Jinyan Huang, Xiaoming Hu, Jianmin Zhang*[*]*, and Ligen Shi*[*]

**This file includes:**

Figure S1

Table S1-8

Note S1

## Supplementary Figures

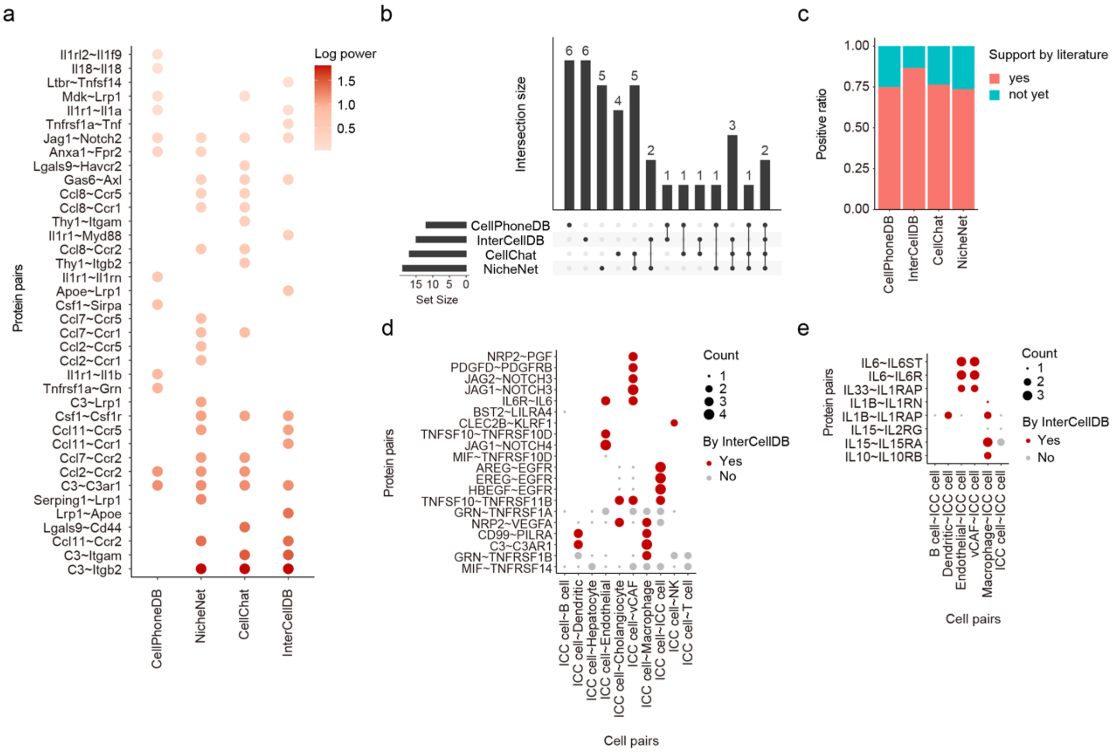

**Figure S1.** Comparison of analysis results between InterCellDB and other tools on human and mouse cases. a to c) Candidate interactions between CAF1 and myeloid cells were evaluated on mouse melanoma scRNA-seq data. a) Predicted protein pairs by any of these methods. Dot color represents the power of protein pairs by multiplying the expression levels of corresponding genes. b) Upset plot showing the count of shared and unique defined protein pairs by these methods. c) Percentage of predicted protein pairs that are supported by literature. d to e) Selected protein pairs were evaluated on human cholangiocarcinoma scRNA-seq data. d) Gene pairs identified by Zhang et al. are reanalyzed using CellPhoneDB, NicheNet, CellChat, InterCellDB. e) interleukin-related gene pairs are reanalyzed using these tools. Dot size represents the number of tools that co-predict the gene pair between corresponding 2 cell groups. Dot color shows whether InterCellDB predicts the specific gene pair.

**Table S1. Introduction of public databases**

| Public databases | NCBI genomes | Ensembl genomes | COMPARTMENTS | Uniprot | GO database | STRING |
|---|---|---|---|---|---|---|
| **Version** | weekly updated version at March 20, 2020 | version 102, 101, 91, 86, 80, 77 | weekly updated version at March 20, 2020 | website resource downloaded at March 20, 2020 | weekly updated version at March 20, 2020 | version 11 |
| **Sources** | https://ftp.ncbi.nih.gov/gene/DATA/GENE_INFO/Mammalia/ | https://www.ensembl.org/, extracted by BioMart | https://compartments.jensenlab.org | https://www.uniprot.org/keywords/, filtered by reviewed records | https://ftp.ncbi.nih.gov/gene/DATA/ | https://version-11-0.string-db.org |
| **Dataset for human** | Homo_sapiens.gene_info.gz | GRCh38 version 102 and GRCh37 | knowledge channel of human data | organisms: human | gene2go.gz limited to taxonomy ID: 9606 | 9606.protein.links.full.v11.0.txt.gz 9606.protein.actions.v11.0.txt.gz |
| **Dataset for mouse** | Mus_musculus.gene_info.gz | GRCm38 version 101, 91, 86, 80, 77 | knowledge channel of mouse data | organisms: mouse | gene2go.gz limited to taxonomy ID: 10090 | 10090.protein.links.full.v11.0.txt.gz 10090.protein.actions.v11.0.txt.gz |
| **Application** | gene reference | mapping proteins to authorized gene names | subcellular location of gene products | molecular function of gene products | functional features of gene products | protein-protein interaction and their action relations |
| **Parameters** | Symbol_from_nomenclature_authority: authorized gene names. type_of_gene: gene function (e.g. protein-coding). | missing proteins in versions not included would be manually collected. | R package GO.db (https://bioconductor.org/packages/GO.db/) was exploited to remap direct GO terms to 13 groups. | keywords were manually merged by functional relevance, and merged types were given. | using all available GO terms | interaction database was further split to 3 sub-databases. annotation on actions was re-collected by action mode and effect. |

**Table S2. Subcellular location of proteins** (*Please see the excel file named Table S2*)

Table S2 contains two sheets of proteins' subcellular location for Human and Mouse. GeneID means unique ID for each gene from NCBI database. GeneName means authorized gene name for each gene from NCBI database. Location means subcellular location of gene-encoded proteins from the COMPARTMENT database. One protein may locate in different cellular regions. And the COMPARTMENT database provided credibility score for each cellular region that one protein may locate in.

**Table S3. Functional feature of proteins (***Please see the excel file named Table S3***)**

Table S3 contains three sheets of proteins' functional feature for Human and Mouse. GeneID means unique ID for each gene from NCBI database. GeneName means authorized gene name for each gene from NCBI database. Type means functional feature of gene-encoded proteins from the Uniprot and GO databases. There are 132 types of proteins' functional feature. We categorized these 132 types of proteins' functional feature into 16 classifications for convenience. The details were presented in the third sheet.

**Table S4. Gene pairs between CAF1 and myeloid cells in mouse melanoma data, predicted by CellPhoneDB, NicheNet, CellChat and InterCellDB. (***Please see the excel file named Table S4***)**

Gene pairs mean potential interaction between CAF1 and myeloid cells by CellPhoneDB, NicheNet, CellChat and InterCellDB. Literature support means whether these gene-encoded protein interactions were supported by previous studies. And we provided the PMID of these literatures. The last four columns mean which method predicted this gene pair.

**Table S5.** Comparison of InterCellDB with previous methods on database composition and intercellular communication analysis.

| | **InterCellDB** | **iTALK** | **CellPhoneDB** | **NicheNet** | **SingleCellSignalR** | **CellChatDB** |
|---|---|---|---|---|---|---|
| **Main database** | | | | | | |
| Species | human, mouse | human | human | human, mouse [a)] | human, mouse [a)] | human, mouse |
| Protein pair category | multiple [b)] | ligand-receptor | ligand-receptor | mostly ligand-receptor | ligand-receptor | multiple [b)] |
| Protein pair count | 5758309, 5882115 | 2649 | 1396 | 12659, 12163 | 3251, 2578 | 1939, 2021 |
| Included gene | 18990, 20938 | 1417 | 979 | 1430, 1359 | 1533, 1287 | 958, 995 |
| **Accessory database** | | | | | | |
| Protein pair annotation | score + action | by ligand type | × | × | × | pathway |
| Protein annotation | location + type + process | × | type | × | × | × |
| **Analysis process** | | | | | | |
| Standardize input | √ | × | × | × | × | × |
| Explore interactions in | √ | × | × | × | × | × |

| | | | | | | | |
|---|---|---|---|---|---|---|---|
| biological context | | | | | | | |
| Evaluate and prioritize cell-cell interactions | score | √ | × | × | × | × | √ |
| | count | √ | √ | √ | × | × | √ |
| Filter significant protein pairs | score | √ | × | √ | √ | √ | √ |
| | probability | √ | × | √ | × | × | √ |
| **Visualization** | | | | | | | |
| Plot on statistical result | | √ | √ | √ | √ | √ | √ |
| Plot with protein attributes | | √ | × | × | × | × | × |
| **Language** | | R | R | Python | R | R | R |

[a] Mouse genes from NicheNet and SingleCellSignalR are not directly provided but can be internally mapped from their orthologous human genes. [b] Multiple protein pair categories include not only ligand-receptor pairs but also receptor-receptor and extracellular matrix–receptor interaction

**Table S6. Action mode for protein pairs**

| Action mode | Explanations |
|---|---|
| Activation | one protein activates another protein, circumstances are:<br>1. bind and activate, e.g. EGF binds and activates EGFR, which causes modification of EGFR structure [1]<br>2. activate remotely, e.g. IL1B activates IL6 as it induces IL6 mRNA up-regulation and promotes expression of IL6 protein [2] |
| Binding | physical association of gene products |
| Catalysis | related to catalytic process |
| Expression | transcriptional regulation, which means one protein affecting the expression of transcription factor of another protein |
| Inhibition | one protein inhibits another protein, which is opposite to 'activation' semantically. The circumstances are: 1. bind and inhibit; 2. inhibit remotely. |
| Ptmod | related to post-translational modification |
| Reaction | related to enzyme reaction, like phosphorylation, ubiquitination, etc. |
| Other | protein interactions not in any of above types |

**Table S7. Action effect for protein pairs**

| Action effect | Explanations |
|---|---|
| Positive | one protein acts on another protein and increases the expression of the latter one |
| Negative | one protein acts on another protein and inhibits the expression of the latter one |
| Unspecified | known action direction of protein-protein interaction, but no evidence for detailed expression change |
| Undirected | both action direction and expression change status are not known |

**Table S8. Proteins shared by iTALK, CellPhoneDB, SingleCellSignalR, NicheNet, CellChatDB, and InterCellDB. (***Please see the excel file named Table S8***)**

This sheet provided all shared mouse proteins from databases of the six methods (ordered by alphabet).

**Note S1: Details on comparison of performance across all methods**

To compare the performance across all methods, we generated a testing protein database that only included those proteins existing in all databases. Then, we applied mostly default settings of every method to generate the results. CellPhoneDB was processed using Python 3.8, and all rest programs were tested using R 4.0.4.

The versions of used packages are given as:

- iTALK, v0.1.0, downloaded from github: https://github.com/Coolgenome/iTALK
- CellPhoneDB, v2.0.0, installed following the detailed steps given in https://github.com/Teichlab/cellphonedb, were used
- SingleCellSignalR, v1.2.0, installed from Bioconductor version 3.12
- NicheNet, v0.1.0, R package is named as 'nichenetr', downloaded from github: https://github.com/saeyslab/nichenetr
- CellChat, v1.1.3, downloaded from github: https://github.com/sqjin/CellChat

***Runtime parameters and settings for iTALK***

The code for running iTALK referred the given example code 'example-code.R' embedded in the package. The top 50% expressed genes for every cell cluster are used. Then, the mouse protein interactions were generated via human-mouse gene orthologs given in Ensembl GRCm39 version 104.

***Runtime parameters and settings for CellPhoneDB***

CellPhoneDB was run using bash scripts. We constructed the required input (count matrix and corresponding metadata) and filtered significant protein pairs ($p$-value < 0.05) from 100 times cell label permutation (by setting --iterations=100). As CellPhoneDB only provided human protein pairs, final results were transformed using human-mouse gene orthologs as that in iTALK.

***Runtime parameters and settings for SingleCellSignalR***

SingleCellSignalR was run with all default settings, and we fetched those gene pairs whose LRscore > 0.5.

***Runtime parameters and settings for NicheNet***

We used NicheNet's ligand activity analysis to generate potential ligand-receptor pairs. The genes of interest in signal receiving cells were set as differentially expressed genes whose *p*-value < 0.05 and $\log_2$ fold change > 0.1, and background genes were collected if over 10% of one cell cluster expressing those genes. Ligands that better predicted genes of interest than background genes (pearson value > 0) were collected. Interactions between those ligands and genes of interest were fetched as result.

***Runtime parameters and settings for CellChat***

We used pre-calculated differentially expressed gene list by Seurat to replace the over expressed genes identified by function 'identifyOverExpressedGenes' provided by CellChat. The average gene expression per cell group was calculated on observations that were 10% trimmed from each end. Interactions whose *p*-value < 0.05 were fetched as result.

***Runtime parameters and settings for InterCellDB***

We used sub-database with highest confidence (combined score ≥ 700) and further select the subset with physically associated ones. Then, 2 protein list were selected, (1) receptor proteins located in plasma membrane; (2) proteins located either in plasma membrane or extracellular region plus proteins annotated as cytokines, growth factors and hormones. We performed network analysis using function 'AnalyzeInterInFullView', and collected those protein pairs with *p*-value < 0.05.

## Supplementary References

[1] E. R. Purba, E. I. Saita, I. N. Maruyama, *Cells* **2017**, *6*.

[2] B. Chen, S. Tsui, T. J. Smith, *J Immunol* **2005**, *175*, 1310.
